# Attitudes toward Animals and Their Welfare among Italian Veterinary Students

**DOI:** 10.3390/vetsci6010019

**Published:** 2019-02-20

**Authors:** Federica Pirrone, Chiara Mariti, Angelo Gazzano, Mariangela Albertini, Claudio Sighieri, Silvana Diverio

**Affiliations:** 1 Department of Veterinary Medicine, University of Milan, 20122 Milan, Italy; federica.pirrone@unimi.it (F.P.); mariangela.albertini@unimi.it (M.A.); 2 Department of Veterinary Sciences, University of Pisa, 56122 Pisa, Italy; angelo.gazzano@unipi.it (A.G.); claudio.sighieri@unipi.it (C.S.); 3 Department of Veterinary Medicine, University of Perugia, 06125 Perugia, Italy; silvana.diverio@unipg.it

**Keywords:** animal welfare, attitude toward animals, veterinary students

## Abstract

As members of the public and the veterinary profession are increasingly concerned about animal welfare, there has been an increased scholarly interest in the attitudes of veterinarians and students toward animals, as these may impact human behavior, which ultimately impacts animal welfare. Here we investigated Italian veterinary students’ demographic data and perceptions about nonhuman animal welfare issues that might be predictive of their attitudes. A survey eliciting information about demographics, knowledge, experience, and perceptions regarding different categories of animals, and including the Animal Attitude Scale (AAS), was administered to undergraduate veterinary medicine students in three Italian universities. Data were analyzed using nonparametric tests, and a value of *p* < 0.05 was considered statistically significant. In total, 876 students completed the questionnaire, with females (75.1%) making up a majority of students in all years of the course. Although veterinary students showed pro-animal welfare attitudes (mean score = 64.20 ± 0.24 out of 100), the findings suggested that year of study, gender, and geographical location had a significant impact (*p* < 0.05). In this study, we found a set of factors that, either individually or combined, help predict a student’s attitude toward animal welfare issues, which will be useful in improving the curriculum strategy in veterinary education in Italy.

## 1. Introduction

Veterinarians have an ethical and professional obligation to respect, safeguard, and promote animal welfare [1]. In Italy and other countries, veterinarians take a professional oath, swearing to use their skills and knowledge for the benefit of animal health and welfare. Attitudes toward animals are important in influencing the way animals are treated [2], and several studies have indicated how a variety of factors, such as gender [3,4], disease state [5], professional discipline [6], perceived responsibility, keeping of a pet, membership in a society [4], and country of residence [7] may influence attitudes and sensitivities of a veterinary practitioner toward animal welfare issues.

Veterinarian attitudes toward animal welfare derive, at least partially, from their training [8,9], and veterinary students are expected to demonstrate a high degree of professional interest in the welfare of animals. Therefore, according to Heleski and others [10], the understanding of veterinary students’ attitudes and perceptions toward these issues is fundamental, as it may be an indirect measure of educational adequacy and effectiveness. However, relatively few studies are available in the scientific literature focusing on veterinary students [11], and they are all cross-sectional studies. For instance, veterinary education has been found to be associated with a lower level of sentience being attributed to animals during the later years of the course and reduced empathy toward animals in male students at two British universities [12]. However, the authors indicated that this change in score could be partially explained by students’ impressions of whether animals experienced boredom. More recently, other studies have underlined the relevance of gender, with a general loss of empathy in veterinary medicine students, especially in their senior years [2,11,13,14,15]. This trend reflected findings in medical students [16]. This decline is concerning and has important implications because veterinarians are positioned as experts in and guardians of animal welfare, which one assumes requires empathy for animals. Besides being important for the sake of animal welfare per se, empathy toward animals may relate to (or be an indicator of) the ability to empathize with humans as well as potentially affect attitudes toward, and treatment of, animals [13].

A rural background and prior experience with farm animals were also associated with a more negative attitude toward animal welfare [11,13]. In our previous study [11], in particular, Italian veterinary students were found to show less concern for the psychological aspects of farm animal welfare compared to pets, with familiarity or willingness to work with livestock being related to less pro-welfare attitudes. Overall, these research findings suggest that attitudes (and what is deemed acceptable treatment) of veterinary students are likely to vary depending on the use of the animal (i.e., companion versus production).

Surprisingly, there is limited evidence about the links between students’ demographic characteristics (other than the gender) and their beliefs and perceptions toward animal welfare. Analyzing these variables in samples of veterinary students might reveal yet uncovered associations, thus offering a better understanding of how future veterinarians perceive different species’ well-being and needs, which should be a precondition for the successful improvement of animal welfare.

The purpose of this study was to explore perceptions of animals, attitudes, and interest in animal welfare, and the possible influence of demographic factors on these perceptions and attitudes in the same sample of Italian veterinary students as in our previous study [11]. With regard to the geographical location of the universities and the students’ place of residence, it is emphasized that an account must be taken of existing differences between Italian regions. Northern Italy, in fact, consists mainly of urban settings, while the central and southern areas are predominantly rural.

## 2. Materials and Methods

### 2.1. Design of Survey

The recruitment of respondents was conducted at the beginning of the academic year 2016–2017 (first week of lectures) in the three Italian veterinary schools where the authors are employed (Pisa, Milan, and Perugia), parallel to and through the same channels as the survey recruitment described previously [11]. Formal education in veterinary medicine is standardized on a national level, so students in the three universities receive similar training in animal behavior and welfare.

All of the students of each course year were given a paper questionnaire in the classroom, which consisted of three sections (see Supplementary Materials Table S1). Briefly, the first section (questions 1–9) contained questions on the participant’s demographic data, such as gender, age, year of course, origin, previous experience with animals, membership in an animal rights association, dietary choices, and preferred employment after graduation. By animal rights association, we meant a voluntary organization protecting animals from cruelty. The second section (questions 10 and 11) focused on the perception of the students about each of the “Five Freedoms” for the protection of livestock and companion animal welfare. This issue was not addressed in this paper, as it was one of the main themes of our previous article [11]. The third section (question 12) contained items that aimed at assessing the students’ attitudes toward nonhuman animals using the 20-item Animal Attitude Scale (AAS) by Herzog et al. [17], as translated and modified by Gazzano [6]. Briefly, the items of the AAS were categorized according to the issue they deal with, and were grouped to create four thematic subscales: Dogs (indicating attitude toward dogs), Food, Research, and Human Moral Dominance. Before completing the questionnaire, students were informed about the aim of the study, anonymity, and that participation in this study was voluntary. Under the requirements of the host institutions, this study did not need ethics approval.

### 2.2. Statistical Analysis

Descriptive statistics were obtained for all variables, and values were reported as means ± standard error (SE). Pearson’s χ^2^ test of independence in 2 × 2 contingency tables was applied to test the association between categorical variables. A one-sample Chi-square test was used to determine the distribution of participants in the “gender” variable. The total scores were calculated, and Cronbach’s alpha was used to estimate internal consistency [18]. For ease of interpretation, the scores were converted into percentage of maximum possible (POMP) scores [19], where 0 and 100 represented the lowest and highest possible scale scores, respectively. Using nonparametric statistics (Mann–Whitney *U* and Kruskal–Wallis with Bonferroni’s correction tests), we tested for differences in the scoring tendency of students. A two-sided *p* < 0.05 was considered to be statistically significant. Statistical analyses were performed while using SPSS version 25.0 (IBM Corp., Armonk, NY, USA).

## 3. Results

A self-selected, convenience sample of 876 students completed the survey (92.6% of questionnaires received, for a total of 946), 173.6 ± 60.1 per year on average.

### 3.1. Participant Characteristics

The demographic characteristics of this sample have been published previously [11]. Briefly, most respondents were female (75.1%) (χ^2^ = 15106.60 on 2 df, *p* = 0.001), aged 22 ± 0.10 years on average, and attended the second year of the course (31.2%). Most of the participants were from central (47.0%) and northern (40.0%) Italy and lived in urban settings (90%). More respondents from the north of Italy were enrolled in the fifth year of the course (56.1%) than in the fourth (44.4%), third (44.6%), second (36.5%), or first year (34.7%) (χ^2^ = 673.73 on 15 df, *p* = 0.001). Similarly, more students attending the Northern University (Milan) were enrolled in the fifth year of the course (54.4%) than in the fourth (38.00%), third (36.9%), second (25.6%), or first year (30.6%) (χ^2^ = 1924.85 on 10 df, *p* = 0.001).

The most common profile for students was to own or to have owned pet dogs and/or cats (42.0%) and to intend to work with either dogs (32.5%) or in mixed practice (e.g., production animals and pets, 34.2%) after graduation. Participants were classified into four self-reported diet groups (see question 8 of the questionnaire): 71.8% meat-eaters, 20.9% vegetarians (eating neither meat nor fish), 2.8% vegans (excluding all animal products), and 4.5% following other types of diets. The vast majority of respondents (88.6%) were not members of any animal rights association.

### 3.2. Animal Attitude Scale Scores

**Figure 1:**
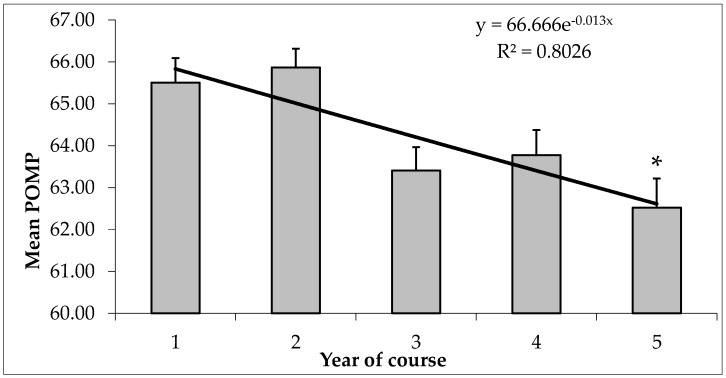
Comparison of mean ± standard error (SE) percentage of maximum possible (POMP) score of the Animal Attitude Scale (AAS) by year of course. Data were represented determining an exponential regression model equation and analyzed using the Kruskal–Wallis test with Bonferroni’s correction (* = significant difference vs year 1 and 2; *p* = 0.001).

**Figure 2:**
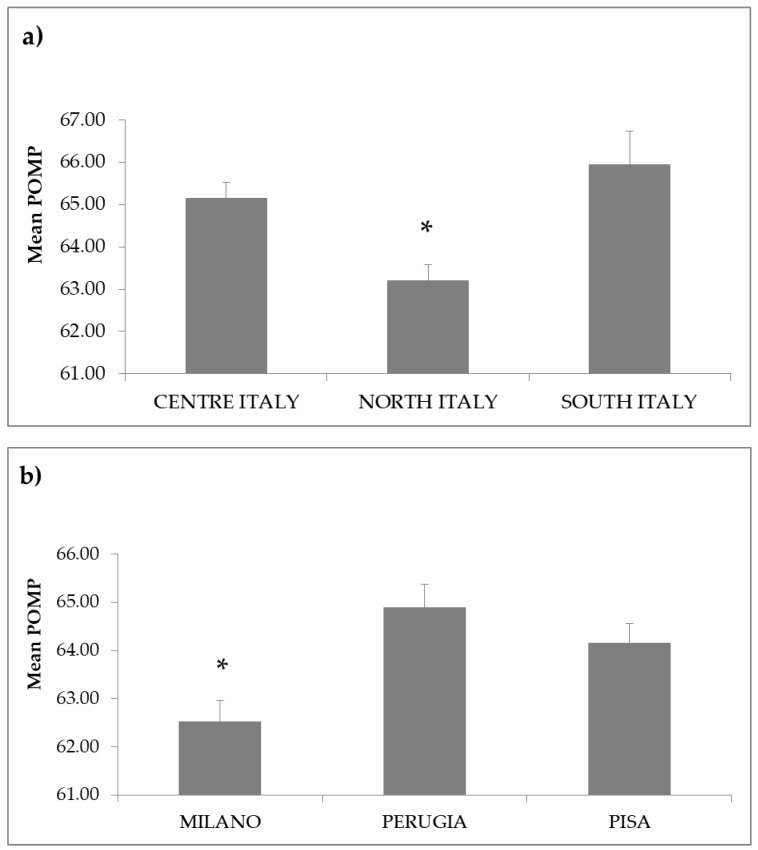
(**a**) Mean ± SE POMP score of AAS by students’ provenance; (**b**) Mean ± SE POMP score by students’ university. Data were analyzed using a Kruskal–Wallis test with Bonferroni’s correction (*p* = 0.001) (* = significant difference vs the other two groups). POMP: Percentage of maximum possible score; AAS: Animal Attitude Scale.

The participants in this study showed a mean POMP score of 64.20 ± 0.24 out of 100 on the AAS, with a good Cronbach’s alpha score of 0.81. The score declined over time (Figure 1), the fifth year presenting the lowest POMP score of the AAS (62.53 ± 0.70, *p* = 0.001). Geographical location also influenced students’ scoring of AAS, as students coming from the north of Italy (63.19 ± 0.39) or attending the Northern University (Milan) (63.85 ± 0.25) showed a significantly lower POMP score (*p* = 0.001) than those from either the south (provenance: 65.95 ± 0.78; university: 64.88 ± 0.49) or center of Italy (provenance: 65.16 ± 0.36; university: 64.14 ± 0.41) (Figure 2a,b).

The AAS scoring tendencies differed significantly also by gender, with females scoring higher than males (66.43 ± 0.28 vs 58.53 ± 0.52, *p* = 0.001).

**Figure 3:**
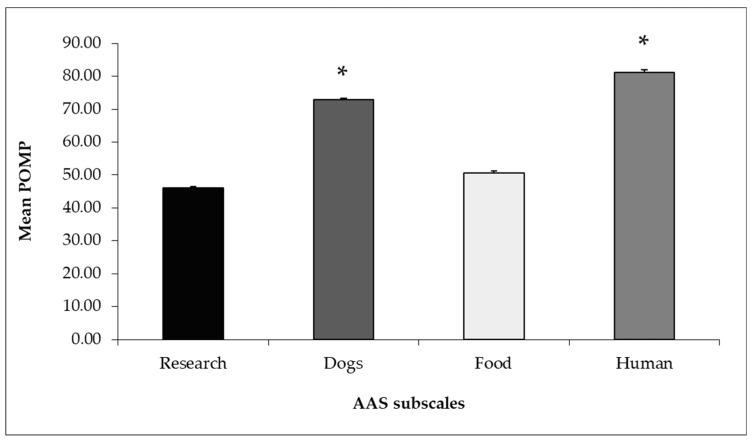
Mean ± SE POMP scores of the four subscales of AAS as suggested by Gazzano et al. [6]. Data were analyzed using a Kruskal–Wallis test with Bonferroni’s correction (*p* = 0.001) (* = significant difference vs Research and Food). POMP: Percentage of maximum possible score.

The mean subscale POMPs were 45% on the Research subscale, 72% on the Dogs subscale, and 51% and 81% on the Food and Human Moral Dominance subscales, respectively (Figure 3).

**Figure 4:**
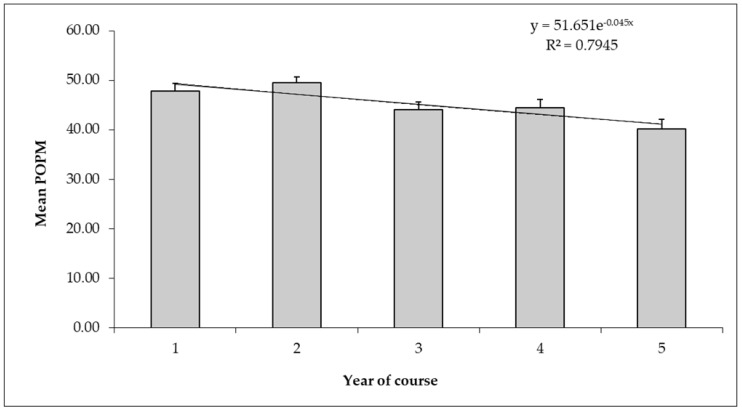
Students’ scoring tendency on animal Research subscale by year of course. Data were represented determining an exponential regression model equation. POMP: Percentage of maximum possible score.

**Figure 5:**
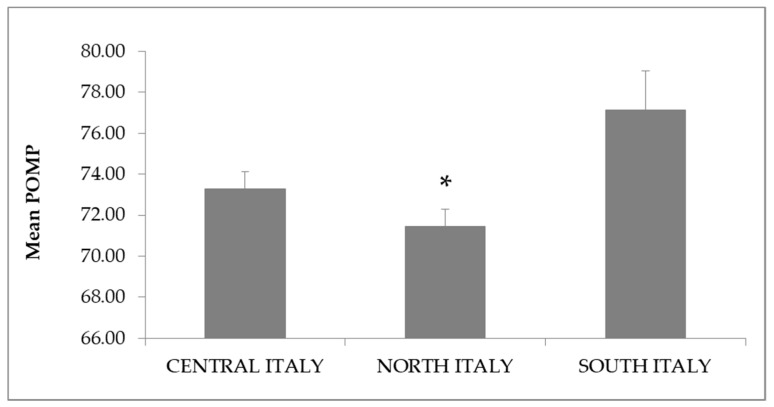
Animal Research subscale’s mean ± SE POMP score by students’ provenance. Data were analyzed using a Kruskal–Wallis test with Bonferroni’s correction (*p* = 0.001) (* = significant difference vs the other two groups). POMP: Percentage of maximum possible score.

Students’ scoring declined over time (Figure 4) on the Research subscale. The effect of a student’s provenance was also detected within this subscale (Figure 5).

Females scored higher than males on the Human Moral Dominance, Food, and Research subscales (*p* = 0.001) (Table 1). No gender difference was observed for the Dogs subscale.

## 4. Discussion

This study explored differences in attitudes to animals and animal welfare issues in Italian veterinary students across gender, year level, geographical provenance, and location of the university attended. The 20-item AAS [17] was used, which is a validated scale to assess general attitudes toward animal welfare issues and other aspects of human–animal interactions. Each of the items is scored on a five-point scale (strongly agree, agree, undecided, disagree, strongly disagree): High scores reflect a greater concern for the welfare of animals. Thus, as suggested by Taylor and Signal [14], higher scores on the AAS indicate a more pro-animal welfare attitude. Overall, the participants in this study showed a mean POMP score of 64.20 ± 0.24 out of 100 on the AAS, corresponding to a total score of 73.77 ± 0.32, which closely resembled the scores emerging from veterinary students in both the U.S. and Europe [14,20]. Although average AAS scores of participants in each course year indicated pro-animal welfare attitudes (possible POMP scores on the AAS range from 0 to 100), student attitudes were likely to change with time spent studying veterinary medicine. Although the effect sizes were quite small (possibly indicating that there may have been confounding factors that could have influenced the observed differences), the students scored exponentially lower on the AAS over time, indicating less of a pro-animal welfare attitude during the later years of the course, particularly the fifth year (POMP score of 62.53 ± 0.70). A similar trend was observed when analyzing the four subscales of AAS: the students’ attitudes toward animal use in research were strongly associated with the year of study, with the score tending to be lower during the later years of the course. This finding might reflect an acceptance of the use of animals in higher year levels of these courses. As this was a cross-sectional study, the differences observed in the attitudes of the students in different years might not necessarily have been related to a shift in the views of individual students. Nevertheless, according to what has been suggested by other authors [12], these data were consistent with a certain degree of hardening or detachment that takes place during veterinary education. Students who have progressed more through a course may therefore counteranthropomorphize animals [21] to cope with the emotional distress they expect to encounter in veterinary work [22]. However, this decline may also reflect the so-called “hidden curriculum” [23]. This locution means a latent space of teaching, antithetical to the formal curriculum, in which medical students build their professional identity based on assumptions and expectations that are not formally conveyed, but are still transmitted within the learning environment. The hidden curriculum is an expression of the deep discrepancy that exists between what is written on study plans in terms of a physician’s professionalism, and how physicians act in a clinical reality, relating to the patient. In the hidden curriculum, the relational, but above all ethical, dimension of the profession falls apart, which results in a progressive decline of moral reasoning during undergraduate training [24], ultimately leading to the formation of a cynical and aloof professional [25]. Veterinarians are among the main stakeholders who may intervene actively to improve the welfare conditions of animals, changing their own behavior. Indeed, veterinary schools have to strengthen formal education on human behavior change (HBC) logic to foster appropriate professional attitudes and ethical practices, together with knowledge and skills for safe and effective practice, among veterinary students.

The respondents in our study were somewhat supportive or neutral about animal experimentation and the eating of food of animal origin, with the scores of these two subscales being 45.97 ± 0.57 and 50.70 ± 0.70, respectively. These attitude scores were also lower in comparison to those obtained on both the Human Moral Dominance and Dogs subscales. We might conclude that different attitudes existed toward the different animal categories, with veterinary students having similarly low attitudes toward animals that humans use for specific purposes. In this regard, we previously found a tendency for this sample of veterinary students to place less importance on the psychological aspects of the welfare of livestock than of companion animals, and to show a low AAS score if they were mostly familiar with, or aspired to work with, livestock [11]. However, according to Van der Weijden [26], a lower score of the students regarding profit/utility species does not necessarily mean that students are not concerned about these animals. Students might just be more concerned about pets and be prepared to do more for their welfare. They might still be concerned about the other animal categories, but just to a lesser extent. In support of this hypothesis, veterinary students in our study scored significantly high on the Human Moral Dominance subscale (POMP 81.20 ± 0.70), showing they were opposed to mere anthropocentric utilitarianism, i.e., the idea that actions are good if they meet the needs of humans, ignoring the benefits or harms to nonhuman individuals [27]. The difference in attitudes experienced by students to different animal use might also be a cognitive strategy adopted to reduce dissonance associated with the paradoxical disconnection between not wanting animals to suffer, yet killing them for food or science [28]. In other words, in order to overcome cognitive dissonance, and the negative affective state associated with it, some students may minimize the sentience of some animals to reduce moral discomfort in their use.

In terms of gender, as expected [11,17], females were significantly more pro-animal welfare than males, as measured by the AAS total and subscale scores. Research exploring the role that gender plays in people’s attitudes toward animals has found that women tend to be more empathic and to have a higher concern for animal welfare compared to men [29,30]. It should also be noted that there were appreciably fewer male than female participants in our study, and this imbalance might be explained by the higher proportion of women being enrolled in veterinary medicine programs in the three courses considered (75.4% Milan, 70.80% Perugia, and 75.70% Pisa).

People’s attitudes about animals have been suggested to be shaped by distinct animal use [31] and opportunities for contact and relationships with animals facilitated by rural and urban places [32]. Studies have shown that people with a rural background have a greater acceptance of animal use than urban people and show greater support for animal experimentation [33,34]. This is due to countries whose populations have a closer relationship with the land and more pragmatic and utilitarian attitudes about animals [33]. Urban people, instead, may never come into contact with the animals they eat and are more likely to keep animals as companions [31], which has been suggested to explain the greater concern for animal welfare [34]. Surprisingly, in our study, coming from the north of Italy, where settings are mainly urban, or attending the Northern University, was associated with less of a pro-animal welfare attitude. However, the majority of these students were enrolled in the fifth year of course: in light of the above considerations, this could, at least partially, explain the result.

## 5. Conclusion

In conclusion, we found an overall quite positive attitude among veterinary students, and we identified student-related factors that may potentially influence their different attitudes toward different categories of animals. It is of note that this was an exploratory data analysis aimed at suggesting and formulating hypotheses that will subsequently be tested by confirmatory data analysis. In fact, a longitudinal study using multifactorial analysis techniques and repeated measurements on each subject is currently ongoing in order to observe whether and how Italian veterinary students’ attitudes really change over time. All of this knowledge will enable educational and training strategies that can be adapted toward successfully motivating pro-animal welfare attitudes and practices in the treatment of all animals through effective formal curriculums that leave no space for the unspoken or implicit.

## Figures and Tables

**Table 1:** Gender differences in subscale POMPs.

Gender		Human Moral Dominance SS POMP	Food SS POMP	Research SS POMP
Female	Mean	83.40	51.85	49.32
	SE	0.52	0.37	0.78
Male	Mean	74.57	47.25	35.35
	SE	1.19	0.66	1.42

POMP: Percentage of maximum possible score. SS: Subscale.

## Supplementary Materials

The following are available online at http://www.mdpi.com/2306-7381/6/1/19/s1, Table S1: Survey Questionnaire.
