# Supplementary material for: Attitudes toward Animals and Their Welfare among Italian Veterinary Students
Source: Vet Sci. 2019 Feb 20;6(1):19. doi: 10.3390/vetsci6010019 (PMC6466256; doi:10.3390/vetsci6010019)
Supplement: Supplementary file 1 [file vetsci-06-00019-s001.pdf]

veterinary sciences

Supplementary Materials: Attitudes toward Animals and Their Welfare among Italian Veterinary Students

Federica Pirrone [1], Chiara Mariti [2,*], Angelo Gazzano [2], Mariangela Albertini [1], Claudio Sighieri [2] and Silvana Diverio [3]

[1] Department of Veterinary Medicine, University of Milan, 20122 Milan, Italy; federica.pirrone@unimi.it (F.P.), mariangela.albertini@unimi.it (M.A.)
[2] Department of Veterinary Sciences, University of Pisa, 56122 Pisa, Italy; angelo.gazzano@unipi.it (A.G.); claudio.sighieri@unipi.it (C.S.)
[3] Department of Veterinary Medicine, University of Perugia, 06125 Perugia, Italy; silvana.diverio@unipg.it
* Correspondence: chiara.mariti@unipi.it; Tel.: +39-328-953-3595

Received: 14 December 2018; Accepted: 31 January 2019; Published: date

Table S1. Survey Questionnaire

Section 1

1. Which year of the course are you attending: ☐ 1 ☐ 2 ☐ 3 ☐ 4 ☐ 5 ☐ Other______

2. Age: ______

3. Gender: ☐ Male ☐ Female

4. Which of the following would best describe your background?

☐ Predominantly rural ☐ Predominantly urban ☐ Predominantly suburban ☐ Other______

5. Where did you mainly live before attending the university?

☐ Italian region ________ ☐ Foreign Country_____

6. With which of the following kinds of animals have you had continued experience (please check all that apply)?

☐ Dog(s) ☐ Cat(s) ☐ Horse(s), Pony(ies) ☐ Cattle, sheep, goat(s) ☐ Pig(s) ☐ Poultry ☐ Small mammals (rabbits, hamsters, etc.) ☐ Cage birds/parrots ☐ Reptiles/amphibians ☐ Other______

7. From the following list, please indicate your preferred type of employment after you graduate from veterinary school: ☐ Farm animals (cattle, pigs, poultry, etc.) ☐ Equine practice ☐ Small animal practice ☐ Mixed practice ☐ Exotic/special species ☐ Other __________

8. Have you ever been on a diet excluding some food of animal origin for ethical reasons (not for health reasons or taste preferences)? ☐ No ☐ Yes, a diet without any product of animal origin ☐ Yes, a diet without meat and fish ☐ Yes, a diet without meat ☐ Yes, other ___________

9. Have you ever been a member of an animal rights association? ☐ No ☐ Yes, which?___________

Section 2

10. In your opinion, how important for the welfare of farm animals are each of the following freedoms listed in the Brambell report in 1965?

	Not at all important	Slightly important	Fairly important	Very important	Extremely important
Freedom from thirst, hunger, and poor nutrition					
Freedom from discomfort (suitable environment)					
Freedom from pain, injury, or disease					
Freedom to express (most) normal behavior					
Freedom from fear and distress					

11. In your opinion, how important for the welfare of pet animals are each of the following freedoms listed in the Brambell report in 1965?

	Not at all important	Slightly important	Fairly important	Very important	Extremely important
Freedom from thirst, hunger, and poor nutrition					
Freedom from discomfort (suitable environment)					
Freedom from pain, injury, or disease					
Freedom to express (most) normal behavior					
Freedom from fear and distress					

Section 3

12. Please indicate your level of agreement/disagreement with each of the following statements:

	Strongly agree	Agree	Undecided	Disagree	Strongly disagree
It is morally wrong to hunt wild animals just for sport.					
I do not think that there is anything wrong with using animals in medical research.					
There should be extremely stiff penalties, including jail sentences, for people who participate in dog fighting.					
Wild animals, such as mink and raccoons, should not be trapped and their skins made into fur coats.					
There is nothing morally wrong with hunting wild animals for food.					
I think people who object to raising animals for meat are too sentimental.					
Much of the scientific research done with animals is unnecessary and cruel.					
I think it is perfectly acceptable for cattle and hogs to be raised for human consumption.					
Basically, humans have the right to use animals as we see fit.					
The slaughter of whales and dolphins should be immediately stopped even if it means some people will be put out of work.					
I sometimes get upset when I see wild animals in cages in zoos.					

In general, I think that human economic gain is more important than setting aside more land for wildlife.					
Too much fuss is made over the welfare of animals these days when there are many human problems that need to be solved.					
Breeding animals for their skins is a legitimate use of animals.					
Some aspects of biology can only be learned through dissecting preserved animals such as frogs.					
Continued research with animals will be necessary if we are to ever conquer diseases such as cancer, heart disease, and AIDS.					
It is unethical to breed purebred dogs for pets when millions of dogs are killed in animal shelters each year.					
The production of inexpensive meat, eggs, and dairy products justifies maintaining animals under crowded conditions.					
The use of animals such as rabbits for testing the safety of cosmetics and household products is unnecessary and should be stopped.					
The use of animals in rodeos and circuses is cruel.					
